# Evidence-Based Classification, Assessment, and Management of Pain in Children with Cerebral Palsy: A Structured Review

**DOI:** 10.3390/healthcare13202608

**Published:** 2025-10-16

**Authors:** Anna Gogola, Rafał Gnat

**Affiliations:** 1 Institute of Physiotherapy and Health Sciences, Academy of Physical Education, 40-065 Katowice, Poland; aniagogola@op.pl; 2 Developmental Neuro-Motor Stimulation Institute International Foundation, 43-600 Jaworzno, Poland

**Keywords:** cerebral palsy, pain, assessment, management, biopsychosocial paradigm

## Abstract

**Highlights:**

**What are the main findings?**

- Pain in children with cerebral palsy is highly prevalent, multifactorial, and often under-recognised, with diagnostic challenges linked to complex underlying mechanisms.
- Evidence supports multimodal, personalised management strategies that combine physical, pharmacological, and psychosocial interventions within an interdisciplinary framework.

**What is the implication of the main finding?**

- Effective pain management requires function-oriented goals, routine screening, and integration of family perspectives to ensure person-centred care.
- There is a pressing need for standardized protocols and high-quality clinical trials to improve evidence-based practice in this field.

**Abstract:**

**Background and objectives**: Pain is a prevalent and often underestimated issue in children with cerebral palsy (CP). When left untreated, pain can result in secondary complications such as reduced mobility and mental health challenges, which negatively impact social activity, participation, and overall quality of life. This review explores the complex mechanisms underlying pain in CP, highlights contributing factors, and places particular emphasis on diagnostic challenges and multimodal pain management strategies. **Methods**: Three scientific databases and, additionally, guideline repositories (2015–2025) were searched, yielding 1335 records. Following a two-step deduplication process, 850 unique items remained. Eighty-five full texts were assessed, of which 49 studies were included. These comprised one randomised controlled trial, 16 non-randomised studies, 12 systematic reviews, 8 non-systematic reviews, and 12 guidelines or consensus statements. Methodological quality was appraised with AMSTAR-2 where applicable, and Oxford levels of evidence were assigned to all studies. **Results**: Study quality was variable: 25% were systematic reviews, with only one randomised controlled trial. This literature identifies overlapping nociceptive, neuropathic, and nociplastic mechanisms of pain development. Classification remains inconsistent, though the International Classification of Diseases provides a useful framework. Only five assessment tools have been validated for this population. Interventions were reported in 45% of studies, predominantly pharmacological (27%) and physiotherapeutic (23%). Evidence gaps remain substantial. **Conclusions**: This review highlights the complexity of pain in children and adolescents with cerebral palsy and the need for a biopsychosocial approach to assessment and management. Evidence supports individualised, multimodal strategies integrating physical therapies, contextual supports, and, where appropriate, medical or surgical interventions. Clinical implementation remains inconsistent due to limited high-quality evidence, inadequate assessment tools, and poor interdisciplinary integration.

## 1. Introduction

Pain is the most common symptom encountered in medicine. It is an unpleasant, subjective sensory and emotional experience [1,2]. Pain is a prevalent and often underestimated issue in children with cerebral palsy (CP). Its reported prevalence ranges from 14% to 76%, with higher rates observed in adolescents, girls, and individuals with severe motor impairments (levels III–V on the Gross Motor Function Classification System—GMFCS) [3,4,5]. Pain becomes a significant issue when it is chronic, of high intensity, and directly impacts quality of life, activity, and social participation [5,6]. This perspective reflects the biopsychosocial paradigm of medicine, which recognises pain as a complex sensory experience influenced by cognitive, affective, and behavioural mechanisms [7].

The International Classification of Functioning, Disability and Health (ICF) provides a valuable framework for understanding the complexity of pain by integrating dimensions of health and disability [8]. By taking into account body functions, activity, participation, personal factors, and environmental influences, ICF enables more comprehensive assessment of pain and its influences, and interdisciplinary treatment strategies [9,10].

It must also be noted that pain identification and assessment in children with CP is highly problematic, particularly for individuals with severe motor impairments who often demonstrate parallel intellectual and/or speech limitations. Traditional pain assessment tools based on verbal communication may be ineffective in this group. Using non-verbal diagnostic techniques, specialists can significantly improve the efficiency of pain assessment and treatment for these individuals [11]. Enhancing communication about pain yields benefits not only in the biological domain but also improves mental well-being and social competence in children with CP [5,12].

Pain diagnosis and treatment in children with CP remains relatively underdeveloped. Early identification and treatment of pain are crucial, since chronic pain stimulation in early life can disrupt maturation of the central nervous system and significantly affect later pain-related behaviours [13,14]. Inadequate analgesia in neonates undergoing surgery or painful diagnostic procedures lowers their pain threshold in later life [15]. Inappropriate treatment of acute pain increases the risk of developing chronic pain conditions. Untreated pain may lead to secondary complications such as reduced mobility and mental health issues [13,16], which negatively affect social activity, participation, and overall quality of life.

Despite extensive research on pain in children with CP, important gaps remain. Prevalence has been well described, but mechanisms—particularly the interplay of motor impairment, cognitive limitations, and psychosocial factors—are less understood. Reliable tools for assessing pain in non-verbal or severely impaired children are still lacking. Although the biopsychosocial model and ICF framework are widely recognised, their translation into routine practice is inconsistent. Research on multimodal strategies is fragmented, and ethical or legal aspects of pain management in this population are rarely explored. These gaps highlight the need for a synthesis that summarises existing evidence and addresses future directions.

Given these numerous pain-related issues, this structured review synthesises current evidence and recommendations for the classification, assessment, and management of pain in children with cerebral palsy, framed within a biopsychosocial perspective and aligned with the ICF. The review briefly outlines the complex mechanisms underlying pain in CP, along with contributing factors. Particular attention is devoted to diagnostic difficulties and multimodal strategies for pain management. In addition, legal and ethical considerations are addressed. This review is informed by clinicians and researchers currently active in the addressed field and presents the challenges and solutions for accurate assessment and management practices, as well as future research directions.

## 2. Materials and Methods

The rationale for adopting a structured review rather than a full systematic review is that the majority of the sources included are themselves systematic reviews or healthcare system-based recommendations from multiple countries, many of which are grounded in systematic review methodology. While these sources have already undergone rigorous validation and represent a high level of scientific evidence, the knowledge is dispersed across numerous publications. A structured review allows for a focused and concise synthesis of this body of evidence, integrating various insights relevant to CP-related. We acknowledge that, unlike a full systematic review, this approach includes limited risk-of-bias appraisal for included sources; however, the methodology was designed to ensure transparent and systematic selection, thereby minimising potential bias.

The literature review was conducted using the PubMed, Scopus, and Google Scholar databases, covering publications in English from 2015 to 2025. This period was selected with respect to the fact that clinical guidelines, in particular, are periodically updated, and sources published more than a decade ago may contain outdated or superseded information. The search employed using of the following phrase: “cerebral palsy” AND (“pain” OR “chronic pain”) AND (“assessment” OR “classification” OR “management”) AND (“evidence-based” OR “evidence-informed”) AND (“review” OR “systematic review” OR “narrative review”).

In addition, supplementary searches were conducted using the websites of relevant professional associations and foundations: American Academy for Cerebral Palsy and Developmental Medicine, European Academy of Childhood Disability (Surveillance of Cerebral Palsy in Europe), Cerebral Palsy Foundation, Cerebral Palsy Alliance; as well as repositories of clinical guidelines recommended by the World Health Organization: National Institute for Clinical Excellence, Australian National Health and Medical Research Council, National Quality Register in Sweden, and Canadian Medical Association Infobase.

The literature search yielded 1310 records from the selected databases and an additional 25 items from website content and guideline repositories. Duplicate records were removed through a two-step process: automated deduplication using Mendeley Reference Manager (v. 2.136.0), which identified duplicates based on title, authors, journal, year, and DOI, followed by manual verification of flagged records to ensure accuracy and retain the most complete version. After deduplication, 850 unique records remained. Titles and abstracts were screened, leading to the exclusion of 765 records as irrelevant to the study topic. The remaining 85 full-text articles were assessed for eligibility, with 36 excluded due to the following criteria: not relevant to the study topic (n = 23), full text unavailable (n = 2), outside the developmental period (n = 5), or addressing other types of pain in children with cerebral palsy (e.g., headache, menstrual; n = 2).

**Figure 1:**
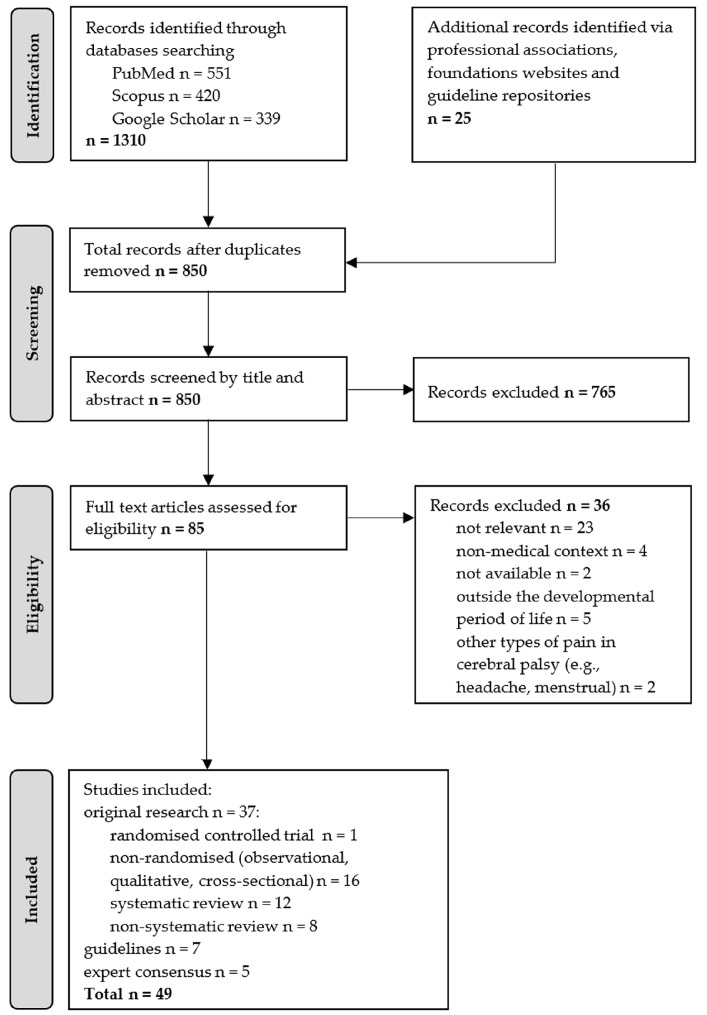
Flow diagram illustrating the process of literature identification, screening, eligibility assessment, and inclusion for the structured review.

A total of 49 studies were deemed eligible for inclusion. Study designs comprised original research articles—including one randomised controlled trial and 16 non-randomised studies (observational, qualitative, cross-sectional)—as well as systematic reviews (with or without meta-analyses; n = 12), non-systematic reviews (narrative or structured; n = 8), clinical practice guidelines (n = 7), and expert consensus statements (n = 5) (Figure 1).

Given the structured design of this review, all eligible studies were included to capture the full current scope of the literature on the topic. Formal methodological quality appraisal assessment was performed where applicable, using AMSTAR-2 for systematic reviews. Oxford levels of evidence were assigned to all included studies to provide an overview of the quality and strength of the evidence.

## 3. Results

This review synthesised evidence from 49 studies on pain assessment, classification, and management in children and adolescents with CP. Study quality was variable: approximately one-quarter were systematic reviews, with only a single randomised controlled trial identified, highlighting the limited availability of high-quality experimental research. Gathered sources indicate that pain in CP arises from overlapping nociceptive, neuropathic, and nociplastic mechanisms, with musculoskeletal pain reported most frequently. Classification remains inconsistent, though the 11th revision of the International Classification of Diseases (ICD-11) provides a useful structure for distinguishing acute, chronic primary, and chronic secondary pain. Assessment is challenging due to developmental variability, communication difficulties, and cognitive impairments; only five tools have been validated specifically in this population. Interventions were described in fewer than half of the studies, primarily pharmacological and physiotherapeutic, with surgical, psychological, and multimodal approaches reported less frequently. The findings emphasise substantial gaps in evidence and the need for targeted research to improve pain evaluation and management in children with CP.

### 3.1. Quality of Included Studies

Of the 49 studies included in the analysis, 12 (24.5%) were systematic reviews (Oxford level 1a) and one (2.0%) was a randomised controlled trial (level 1b). Non-randomised observational and cross-sectional studies (level 2b) accounted for 12 (24.5%), while three (6.1%) were qualitative studies (level 2c). The remainder comprised 8 non-systematic reviews (16.3%), 7 clinical guidelines (14.3%), and 5 expert consensus studies (10.2%), all classified as level 5 evidence.

Methodological appraisal of systematic reviews using AMSTAR-2 identified 7 (14.3% of all studies; 58.3% of systematic reviews) as high quality and 5 (10.2% and 41.7%, respectively) as moderate quality.

Notably, some level 5 sources incorporated higher-level evidence; for instance, one non-systematic review [13]^5^ (superscript indicates the Oxford level of scientific evidence) included 16 studies of level 2b quality.

### 3.2. Characteristics of Study Populations: Age, Cerebral Palsy Subtypes

Across the 49 included studies, 17 (34.7%) investigated children aged 0–12 years, 6 (12.2%) focused on adolescents aged 13–18 years, and 26 (53.1%) addressed combined cohorts of children and adolescents.

With regard to cerebral palsy subtypes, 7 studies (14.3%) examined spastic CP, 3 (6.1%) dyskinetic CP, and 5 (10.2%) mixed presentations, while the majority (n = 34; 69.4%) did not explicitly specify the CP subtype investigated.

### 3.3. Pathophysiology of Pain

Regarding the current understanding of the pain pathophysiology, the reviewed sources indicate that pain in individuals with CP arises from a combination of nociceptive, neuropathic, and nociplastic mechanisms [17]^5^ (non-systematic review); [18]^2b^ (non-randomised study); [19]^2b^ (non-randomised study) (Table 1). The relative contribution of each mechanism may vary according to the type and severity of CP. Understanding these patterns provides a basis for targeted pain assessment and management.

Using this framework, 26 of the 49 included studies (53.1%) specifically addressed at least one of the specified pain types. Of these, 8 studies (30.8%) focused on nociceptive pain associated with tissue injury or procedural interventions, 5 (19.2%) examined neuropathic pain, such as that arising from nerve injury or dystonia, and a further 5 (19.2%) investigated nociplastic pain, encompassing central mechanisms linked to altered nociception and chronic pain. The remaining 8 studies (30.8%) considered mixed pain types, typically covering both acute and chronic mechanisms.

### 3.4. Pain Classification

The sources reviewed indicate that effective pain management in children with CP requires precise identification and understanding of the underlying mechanisms of pain, encompassing biological, anatomical, and physiological factors, as well as psychosocial influences [13]^5^ (non-systematic review); [26]^1a^ (systematic review). The consistent application of systematic pain classification holds considerable potential to enhance treatment strategies tailored to the individual needs of each child.

Pain classification within the CP population remains complex and inconsistent. In some instances, categorisation is based on aetiology; in others, on temporal features such as pain duration, or on additional determinants. Furthermore, certain pain presentations may legitimately fall into more than one category [27]^5^ (non-systematic review).

Referring to the ICD-11, two of the sources analysed [27]^5^; [28]^5^ (both expert consensus) emphasise that paediatric pain should be classified not only by aetiology but also by duration. The types of pain identified are presented in Table 2. According to ICD-11, acute and chronic pain are distinguished, with chronic pain further subdivided into chronic primary pain, defined as pain not attributable to another medical condition, and chronic secondary pain, where pain arises as a consequence of an identifiable underlying disorder.

Some studies indicate that musculoskeletal pain is the most frequently reported pain in children with CP. It was identified in 41.5% of the studies analysed by Vinkel et al. [27]^5^. This type of pain is usually categorised as chronic secondary pain, as several contributing factors (e.g., muscle spasms, subluxations, scoliosis, osteoporosis, joint malalignment) play a role in its development. However, establishing a direct relationship between musculoskeletal abnormalities and reported pain symptoms remains challenging. Consequently, the clinical presentation may often reflect overlapping features of different mechanisms, making chronic primary pain a more plausible classification in many cases.

Acute pain is identified two times less frequently, i.e., in approximately 20% of the studies [27]^5^, though only one study specifically distinguished between acute and chronic forms. In this group, the most frequently described subtypes were procedural and postoperative pain, both of which were classified as secondary pain driven by nociceptive mechanisms. Notably, acute pain associated with medical procedures, surgery, and rehabilitation has gained increasing recognition in clinical practice, and these conditions are also commonly placed within secondary pain categories. Comparable findings have been reported in other studies [22]^5^ (non-systematic review); [23]^2b^ (non-ransomised study); [29]^2c^ (non-ransomised study).

Another classification considers the location of pain: lower limbs (32–82%), upper limbs (4–19%), abdomen (11–32%), head (10–30%), back (9–25%) [5]^1a^ (systematic review); [30]^2b^ (non-randomised study). Additional pain areas reported in the literature, though not directly related to CP, include non-specific lower back pain, abdominal pain, dental pain, menstrual pain, and headaches [29]^2c^ (non-randomised study).

### 3.5. Pain Assessment Tools

As with classification, pain assessment in children is regarded as a challenging issue. Literature indicates that in early life, pain expression is significantly limited. Commonly recognised signs—such as increased motor activity, crying, facial expressions, increased heart rate, or elevated blood pressure—may be limited or not manifested at all, depending on the individual’s overall condition. These symptoms may also be caused by other factors such as discomfort, cold, or hunger [31]^2c^ (non-randomised study).

Over time, children develop a broader range of expression, culminating in verbal communication. However, in children with disabilities, this developmental process is often disrupted–either due to motor limitations or coexisting intellectual impairments. Therefore, assessing pain in children with CP frequently requires the involvement of an augmentative and alternative communication specialist [32]^1a^ (systematic review); [33]^5^ (expert consensus); [34]^5^ (expert consensus); [35]^5^ (guideline).

Pain intensity in children is shaped by a range of additional factors, including age, emotional state, and, in particular, anxiety associated with hospitalisation, prior pain experiences, and previous medical interventions. The literature frequently highlights the importance of regularly measuring and assessing pain in paediatric patients [36]^5^ (guideline); [37]^5^ (guideline); [38]^5^ (non-systematic review); [39]^5^ (guideline); [40]^5^ (guideline). The most comprehensive analysis of pain assessment tools in the CP population is the non-systematic review by Harvey et al. [13]^5^. This review categorises available tools into two principal groups: (1) patient-reported measures, or self-assessment tools, and (2) observer-reported measures, in which pain is evaluated by someone other than the patient. While patient-reported measures are generally regarded as more informative, their applicability is limited by the child’s age and cognitive capacity. It should be emphasised that only five of the pain assessment tools reviewed by Harvey et al. [13]^5^ have undergone direct validation in individuals with CP (Table 3).

The choice of an appropriate tool should be individualised, acknowledging the varying pace of children’s developmental trajectories [33]^5^ (expert consensus); [41]^1a^ (systematic review); [42]^5^ (expert consensus).

**Table 3:** Pain assessment tools for children with cerebral palsy, categorised by type and purpose, [13] (references [11,43,44,45,46,47,48,49,50,51,52,53,54,55,56,57]) all Oxford level 2b (non-randomised studies), were originally quoted in [13] as Oxford level 5 (non-systematic review).

Tools	Pain-Related Limitations	Coping Mechanisms for Pain
Patient- reported	Bath Adolescent Pain Questionnaire [43] Child Activity Limitations Interview [44] Pain Interference Index [45] Modified Brief Pain Inventory * [46] Patient-Reported Outcome Measurement Information System [47]	Bath Adolescent Pain Questionnaire [43] Cerebral Palsy Quality of Life-Teen * [48] Child Self-Efficacy Scale [49] Fear of Pain Questionnaire for Children [50] Fear of Pain Questionnaire for Children —Short Form [51] Pain Catastrophizing Scale [52] Pain Coping Questionnaire [53] Pain Coping Questionnaire Short Form [54] Paediatric Pain Coping Inventory [55]
Observer-reported	Patient-Reported Outcome Measurement Information System Paediatric Proxy Pain Interference Scale [47] Bath Adolescent Pain Questionnaire for Parents [56] Modified Brief Pain Inventory-Proxy * [46]	Fear of Pain Questionnaire Parent Version [50] Pain Catastrophizing Scale [52] Pain Coping Questionnaire Parent Version [54] Paediatric Pain Coping Inventory Parent Version [55]
Observational instruments	Paediatric Pain Profile * [11] The Non-communicating Children’s Pain Checklist—Revised * [57]	Not applicable

* validity evaluated in patients with cerebral palsy.

Among the 49 studies included in our review, pain intensity was the parameter most frequently assessed. It was evaluated in 13 studies (26.5%), most commonly through numerical or visual rating scales. In addition, two studies (4.1%) employed the Paediatric Pain Profile.

### 3.6. Pain Management

Out of the 49 studies reviewed, 22 (44.9%) reported interventions targeting pain reduction in children with cerebral palsy. Pharmacological approaches were the most frequently described, appearing in 6 studies (27.3%), followed by physical or physiotherapeutic methods in 5 studies (22.7%) and surgical techniques in 4 studies (18.2%). Psychological or psychosocial interventions were reported in 3 studies (13.6%), while multimodal or interdisciplinary strategies were identified in 2 studies (9.1%). An additional 2 studies (9.1%) explored other approaches, including biofeedback, cognitive coping strategies, and family education.

Effective pain management in children begins with identifying and understanding the underlying mechanisms of pain as well as the modifying factors, including the psychosocial domains of the ICF framework. There remains a noticeable lack of high-quality research on effective pain treatments specifically for children with CP [6]^1a^ (systematic review); [23]^2b^ (non-randomised study).

The primary goal of pain management in children is complete pain relief, or, if that is not possible, reducing pain intensity to an acceptable level. Proper analgesia in paediatric patients is an essential component of Enhanced Recovery After Surgery protocols in paediatric care. These protocols require regular pain assessments at least three times per day, with documentation of the results alongside other vital signs in the patient’s medical record [36]^5^ (guideline); [58]^5^ (non-systematic review). In many cases, it is not possible to clearly distinguish between causal and symptomatic treatments. Attention should also be given to minimise the discomfort associated with pain relief procedures [59]^5^ (guideline); [60]^1a^ (systematic review); [61]^5^ (sguideline) (Table 4).

### 3.7. Legal Aspects of Pain Management

Occasionally, the sources analysed indicate that pain management in children with cerebral palsy encompasses certain legal considerations. The duty to alleviate pain stems from a fundamental human right—the right to relief from suffering. The XIII World Congress on Pain Declaration (Montreal Declaration) highlighted that this right is especially pertinent to children ([76]^5^ expert consensus).

## 4. Discussion

This review synthesises evidence on the classification, assessment, and management of pain in children with CP within a biopsychosocial and ICF framework. It considers the heterogeneous mechanisms underlying pain, the particular diagnostic challenges posed by motor and communication impairments, and the need for multimodal management strategies. Drawing on both clinical and research perspectives, the review highlights current challenges while outlining opportunities and priorities for future work.

### 4.1. Quality of Included Studies

The overall quality of the included studies was mixed, reflecting both strengths and limitations of the current evidence base on pain in children with CP. While nearly one quarter of the studies were systematic reviews and a small number represented randomised controlled trials or observational designs, a considerable proportion relied on lower levels of evidence such as non-systematic reviews, clinical guidelines, or expert consensus. Methodological appraisal indicated that most systematic reviews were of high or moderate quality, providing a solid foundation for evidence synthesis. However, the predominance of lower-level sources highlights a reliance on expert opinion and narrative reviews, underscoring the need for more robust primary research. Some lower-level sources incorporated higher-quality data, but this does not substitute for rigorous trial evidence. Study quality was not applied as a selection criterion, as our aim was to synthesise the entirety of available knowledge in this narrow field. Future work should prioritise high-quality, methodologically sound studies, the development of validated assessment tools, and interdisciplinary approaches to enhance both understanding and management of pain in children with CP.

### 4.2. Classification of Pain

Pain in children with CP is a complex, multidimensional phenomenon that rarely reflects a single mechanism. One of the most influential frameworks for understanding pain in this population is the ICD-11-based system proposed by Vinkel et al. [27], which distinguishes acute from chronic pain. Chronic pain is further subdivided into primary pain, which is not attributable to another medical condition, and secondary pain, which arises from an identifiable underlying disorder. Mechanistically, pain may be nociceptive, resulting from tissue damage or inflammation; neuropathic, caused by nerve injury; or nociplastic, reflecting altered central nociception and sensitisation, often accompanied by fatigue, sleep disturbances, and cognitive difficulties [17]. Evidence indicates that pain in CP often arises from the interaction of excessive nociception, central sensitisation, and psychosocial factors [22,77]. Most research to date has focused on nociceptive pain, while neuropathic and nociplastic mechanisms remain underexplored [13,21]. Acute and chronic pain are frequently conflated, despite reports that approximately two-thirds of children with CP experience acute pain and one-third chronic pain [23]. Individuals with dyskinetic CP have been shown to carry a 3.5-fold increased risk of chronic pain [21].

Understanding these distinctions has important clinical implications. For instance, recognising nociplastic mechanisms may prioritise non-pharmacological interventions, whereas neuropathic pain may respond preferentially to specific pharmacological approaches. The complex interplay of modulating factors—including psychosocial and cognitive dimensions—emphasises the need for interdisciplinary assessment and management. However, current practice often places disproportionate emphasis on biological factors, with limited integration of the biopsychosocial model and the ICF framework. Future research should focus on validating multidimensional, mechanism-informed classification tools that support tailored clinical strategies, bridging the gap between conceptual frameworks and applied pain management in children with CP.

### 4.3. Pain Assessment

Assessment of pain in children with CP remains a substantial clinical and methodological challenge due to communication difficulties, cognitive impairments, and the heterogeneity of motor function within this population. As summarised in Table 3, a wide range of paediatric pain assessment instruments is available; however, the majority have not been validated for use in children with CP. Kingsnorth et al. (2015) identified only seven of 52 chronic pain tools as relevant to this group [26], while Breau et al. (2002) emphasised the scarcity of appropriately validated instruments [57]. Among validated instruments, the Paediatric Pain Profile [11] can be considered the most adequately validated tool for children with CP, demonstrating robust psychometric properties and clinical feasibility, particularly for non-verbal individuals and those using augmentative and alternative communication [11,32].

In clinical practice, the choice of assessment tool should be guided by the child’s communicative and cognitive abilities. For verbal children with mild to moderate motor impairment, self-report measures such as the Modified Brief Pain Inventory or Cerebral Palsy Quality of Life-Teen are appropriate and allow concurrent evaluation of pain intensity and functional interference. For non-verbal children or those with severe intellectual disability, observer-based instruments such as the Paediatric Pain Profile or the Non-communicating Children’s Pain Checklist—Revised provide more reliable and clinically meaningful information. Integrating caregiver observations with structured professional assessment appears to offer the most comprehensive approach to capturing the multifaceted nature of pain in this population.

Despite these developments, the existing literature remains fragmented and predominantly descriptive. Most studies assess pain intensity in isolation, with limited consideration of interference, emotional burden, or coping mechanisms. The absence of standardised and multidimensional assessment frameworks hinders comparison across studies and limits the applicability of findings in clinical settings.

Future research should prioritise the rigorous validation of multidimensional assessment tools that reflect the specific communication and functional profiles of children across all levels of the GMFCS. Development of augmentative and alternative communication—compatible or digital assessment platforms may enhance accessibility and clinical utility. Co-design approaches, incorporating children, families, and multidisciplinary teams, should be encouraged to ensure that new instruments are both psychometrically robust and clinically relevant.

### 4.4. Pain Management

Despite the high prevalence of pain in children with CP, consensus on effective management remains lacking, rendering pain one of the least understood and studied problems in this population [6]. Clinical manifestations are heterogeneous, and the complex pathophysiology complicates guideline development. Pain relief is often secondary to addressing motor impairments, and aside from identifiable causes [22]. Coping strategies may mitigate interference with daily functioning, but standardised evaluation and integration into management plans are inconsistent.

Overall, substantial gaps persist in high-quality evidence for pain management in CP. This review offers a synthesis of evidence within a biopsychosocial and ICD-11-informed framework, emphasising mechanism-specific, individualised, and multidisciplinary approaches. Future research should prioritise mechanism-specific, multimodal interventions, standardised outcome measures, and longitudinal evaluation to guide clinical practice and improve quality of life in this population.

#### 4.4.1. Pharmacological Options

Pain management in CP may be complicated by interactions with medications for spasticity and epilepsy, and children with neurological impairments may be more susceptible to side effects [22]. Pharmacological therapy should be guided by thorough assessment, delivered within a multidisciplinary framework, and subject to regular review to ensure therapeutic benefit outweighs risk [59]. Evidence from reviews and guidelines supports pharmacological interventions as first-line strategies for chronic pain [5,6,23,35,36,59,60]. Intrathecal baclofen demonstrates moderate-to-high quality evidence for hypertonia-related pain and postoperative analgesia [6], while botulinum toxin may reduce hypertonia-associated pain in non-ambulant children (GMFCS IV–V) [64]. Gabapentin (37%) and oral baclofen (33%) are frequently prescribed for dystonia-related pain, though high-quality efficacy data remain limited [62]. Acute and procedural pain require distinct management strategies. Multimodal analgesia—combining agents acting via different mechanisms—is a fundamental principle [36,78]. Opioids, primarily μ-receptor agonists, are used for moderate to severe pain but carry risks including sedation, respiratory depression, constipation, and paradoxical hyperalgesia [36]. Non-opioid analgesics, including paracetamol, metamizole, NSAIDs, and COX-2 inhibitors, provide analgesic, antipyretic, and sometimes anti-inflammatory effects, either alone or within multimodal regimens, allowing a reduction in opioid doses [36]. Regional anaesthesia provides superior, longer-lasting analgesia and reduces opioid requirements

#### 4.4.2. Physical and Non-Pharmacological Therapies

Reports regarding pain associated with physiotherapy in children with CP are conflicting. Some authors note that certain physiotherapy interventions, particularly assisted or passive stretching and joint mobilisation, may provoke pain [25,65,68,79]. In contrast, Ostojic et al. (2019) found no direct evidence identifying the causes of procedural pain during physiotherapy [6]. Rather than discontinuing these interventions, adaptation of equipment and prioritisation of less painful techniques are recommended [66]. The majority of authors regard physical therapies—including physiotherapy, massage, hydrotherapy, therapeutic positioning, and the use of assistive devices—as essential for addressing musculoskeletal contributors to pain, preserving mobility, and supporting functional capacity, particularly when musculoskeletal complications underlie chronic discomfort [35,66,80]. Exercise is also promoted in national guidelines from NICE (UK) [35,61], the New South Wales Ministry of Health [39], and the Scottish Government [59], all of which advocate for the role of early intervention in alleviating pain. These guidelines emphasise the importance of encouraging movement, increasing physical activity, and restoring functional ability as essential components of pain management in children with CP.

#### 4.4.3. Surgical Treatment

In children with severe dyskinetic symptoms and central pain mechanisms, deep brain stimulation (DBS) may offer a precision-targeted intervention when conventional treatments fail. Perides et al. (2020) reported significant reductions in dystonia and pain severity in selected paediatric cases [70]. However, systematic reviews by Shaheen et al. (2023) [71] and Frizon et al. (2020) [72] found insufficient evidence to support DBS efficacy, although no adverse events were reported.

Beyond DBS, a range of orthopaedic and neurosurgical interventions targeting secondary musculoskeletal pathologies can contribute to pain reduction. Procedures such as tendon lengthening, soft-tissue releases, corrective osteotomies, and spinal or hip surgeries are commonly performed to address contractures, malalignment, and deformities, which are frequent sources of chronic secondary pain in children with CP [6,22,35]. While these interventions primarily aim to improve function and posture, they often have the additional benefit of alleviating pain associated with musculoskeletal strain or joint malposition. The choice of surgical approach should be individualised, based on the child’s pain profile, functional goals, and severity of musculoskeletal complications, and integrated into a multidisciplinary care plan with postoperative rehabilitation.

#### 4.4.4. Psychological Support and Other Techniques

Psychological support in paediatric pain management encompasses several distinct approaches [74], all of which have clear relevance for children with CP. Cognitive-behavioural interventions emphasise skills training, cognitive coping strategies, and parental involvement, and are among the most consistently effective psychosocial approaches for reducing pain-related distress and improving functional outcomes [75]. Distraction technique—including games, videos, and guided imagery—are widely used to alleviate procedural pain such as needle-related distress and postoperative discomfort [73,74,81]. Hypnosis, relaxation, and related strategies have also demonstrated benefit in acute pain contexts [73]. Importantly, family education and active involvement enhance the effectiveness of these methods, as parents frequently shape the child’s coping responses and engagement.

Interdisciplinary programmes integrating these interventions, sometimes delivered through remote or blended formats, are increasingly reported [75]. However, despite promising evidence, psychosocial strategies remain underutilised in CP care, where pharmacological and biomedical interventions often dominate. Their stronger integration into clinical pathways is particularly important within the biopsychosocial model, given the well-documented role of psychological and social factors in amplifying or mitigating pain. Strengthening the evidence base, especially through CP-specific validation and long-term outcome studies, will be critical to ensuring these approaches are systematically embedded alongside physical and pharmacological therapies. Finally, psychosocial interventions may be particularly valuable for addressing nociplastic mechanisms of pain, where central sensitisation and emotional distress interact, underscoring the need for interdisciplinary strategies that target both sensory and psychosocial dimensions of pain.

### 4.5. Limitations

This review has several limitations. The included studies were heterogeneous regarding design, population characteristics, pain assessment methods, and reported outcomes, which limits generalisability and precludes meta-analysis. Most studies were observational or non-randomised, with only one randomised controlled trial, and systematic reviews varied in methodological quality, resulting in an overall limited evidence base. Publication bias is possible, and the search was restricted to English-language studies, potentially excluding relevant data.

Additionally, study quality was not used as an inclusion criterion to allow a broad synthesis of this relatively narrow field, meaning some findings were derived from lower-quality sources. Reporting in the included studies was often incomplete, particularly for pain mechanisms, functional outcomes, and intervention protocols.

## 5. Conclusions

This structured review highlights the multifaceted nature of pain in children and adolescents with cerebral palsy and underscores the importance of a biopsychosocial approach to its assessment and management. Evidence supports multimodal strategies tailored to individual needs, combining physical therapies, environmental and contextual supports, and, where appropriate, medical or surgical interventions. Despite this, clinical implementation remains inconsistent, constrained by limited high-quality evidence, inadequate assessment tools, and insufficient interdisciplinary integration. Pain should be recognised not merely as a symptom but as a factor impacting activity, participation, emotional well-being, and overall development—central domains of the ICF framework. Optimising care requires function-oriented goals, systematic screening, and collaborative, interdisciplinary approaches. Critically, incorporating the perspectives of children and their families is essential to ensure person-centred, meaningful interventions. This review emphasises the need for future research to develop validated assessment tools, clarify mechanism-specific management strategies, and integrate biopsychosocial principles into routine clinical practice.

## Figures and Tables

**Table 1:** Summary of the pathophysiological mechanisms contributing to pain in children with cerebral palsy. The table outlines the principal types of pain (according to the International Association for the Study of Pain), underlying mechanisms, characteristic clinical features, and factors modulating pain perception in this population.

Pain Type	Mechanism	Key Features
nociceptive pain [19]^2b^	- tissue damage or potential damage	- muscle spasms (especially in spastic CP) - joint deformities due to spasticity-related misalignment - soft tissue strain from abnormal posture and movement patterns
neuropathic pain [17]^5^, [19]^2b^, [20]^5^	- nervous system damage or dysfunction	- central pain pathway dysfunction (abnormal processing of nociceptive signals in the CNS) - peripheral nerve damage (e.g., neuropathy)
nociplastic pain [17]^5^, [19]^2b^	- altered central nociception without evident tissue injury - dysfunctional central pain processing	- central sensitisation (increased excitability of nociceptive neurons in the CNS) - widespread pain without clear somatic cause - associated with CNS-related symptoms such as fatigue, gastrointestinal dysfunction, sleep disturbances, cognitive impairment - conditions such as psychogenic pain, fibromyalgia, bladder pain syndrome, headaches (including migraines)
modulating factors	- factors influencing pain perception	- severity of hypertonia (spasticity, dystonia [18]^2b^, [21]^2b^, [22]^5^) - functional mobility level (GMFCS III–V) [23]^2b^, [24]^2b^ - psychosocial factors (emotional state, stress, social environment) [25]^2b^

CP—cerebral palsy; CNS—central nervous system; GMFCS—gross motor function classification score; superscript indicates the Oxford level of evidence.

**Table 2:** Classification of pain types and their prevalence [27]^5^ (non-systematic review) in children with cerebral palsy.

Pain Type	Cause	Prevalence (%)
Acute pain	*Procedural pain*	
	- botulinum toxin A injections	5.6
	- surgical procedures	1.5
	- other causes	0.5
	*Postsurgical pain*	8.2
	*Rehabilitative pain*	
	- physiotherapy procedures	2.6
	- standing frame use	1.5
Chronic primary pain	Complex regional pain syndrome	0.5
Chronic secondary pain	*Musculoskeletal pain*	
	- deformities and misalignment	20.5
	- spasticity and dystonia	13.8
	- bone pain	1.0
	- other	3.1
	*Visceral pain*	
	- gastrointestinal pain	1.5
	*Neuropathic pain*	
	- peripheral neuropathic pain	1.0
	- central neuropathic pain	0
	- postsurgical	0.5
Non-specific pain	- quality of life, mental health, and coping	20.5
	- other	9.7

**Table 4:** Recommended multimodal pain management approaches in children with cerebral palsy, including pharmacological, physical, surgical, psychological, and complementary strategies.

Type of Intervention	Approach	Details
Pharmacological treatment	Multimodal analgesia	Non-opioid analgesics as first-line; opioids when necessary [6]^1a^ **, [36]^5^, [62]^2b^
	Analgesics	Paracetamol, metamizole, NSAIDs, corticosteroids [6]^1a^ **, [35]^5^, [36]^5^, [63]^1a^ **
	Co-analgesics	Lidocaine, alpha-2 receptor agonists, ketamine, gabapentinoids, magnesium [4]^2b^, [13]^5^, [36]^5^, [37]^5^, [63]^1a^ **
	Regional anaesthesia	Regional blocks with/without alpha-2 [6]^1a^ **, [36]^5^, [37]^5^, [64]^1a^ **
	Muscle spasm management	Baclofen (oral, intrathecal), botulinum toxin (note injection discomfort) [6]^1a^ **, [13]^5^, [22]^5^, [35]^5^, [62]^2b^
Physical and non-pharmacological therapies	Physiotherapy	Regular physiotherapy for mobility, strength, flexibility; massage, aquatic therapy, assistive devices [61]^5^, [65]^2c^, [66]^1a^ *
	Activity	Encouragement of physical activity (challenging in severe disability) [35]^5^, [39]^5^, [59]^5^, [67]^2b^
	Additional interventions	Positioning, stretching, massage, heat/cold therapy, rest, breathing exercises, hydrotherapy [23]^2b^, [68]^2b^, [69]^2b^
	Surgical options	Deep brain stimulation for dystonic CP [70]^2b^, [71]^1a^ *, [72]^1a^ *
Surgical treatment	Surgical interventions	Indicated for severe pain due to contractures or joint malalignment; last resort [70]^2b^
Psychological support and other techniques	Psychological therapies	CBT, psychological support [36]^5^, [73]^1b^, [74]^1a^ *
	Complementary techniques	Distraction, visualisation, education [73]^1b^, [75]^5^

NSAIDs—non-steroidal anti-inflammatory drugs; CBT—cognitive-behavioural therapy; CP—cerebral palsy; ** high and * moderate methodological quality according to AMSTAR-2 (only for systematic reviews); superscript indicates the Oxford level of evidence.

## Data Availability

No new data were created or analysed in this study.

## Abbreviations

CP	Cerebral Palsy
ICF	The International Classification of Functioning, Disability and Health
GMFCS	Gross Motor Function Classification System
CBT	Cognitive-Behavioural Therapy
DBS	Deep Brain Stimulation
ICD-11	11th edition of the International Classification of Diseases
NSAIDs	Non-steroidal anti-inflammatory drugs
